# Correlation of Pattern of Invasion, Stromal Inflammation and Lymphovascular Invasion With Histopathological Grading in Oral Squamous Cell Carcinoma: A Retrospective Study

**DOI:** 10.7759/cureus.52233

**Published:** 2024-01-13

**Authors:** Samiha J Khan, Madhuri Gawande, Alka H Hande, Swati K Patil, Archana M Sonone

**Affiliations:** 1 Oral Pathology and Microbiology, Sharad Pawar Dental College, Datta Meghe Institute of Higher Education and Research, Wardha, IND

**Keywords:** histopathology (hp), overall survival (os), tumor front, inflammation, metastasis, pattern of invasion, squamous cell carcinoma, prognosis

## Abstract

Introduction: Despite the progress in diagnostics and treatment modalities, the survival rate of oral squamous cell carcinoma (OSCC) patients has remained unchanged. Early diagnosis of the disease helps in better treatment and prognosis. Identifying clinicopathological and histopathological parameters that help predict disease progression is crucial.

Objectives: To assess the significance of various clinical and histopathological factors in OSCC and to correlate the patterns of invasion of tumour (POI), stromal inflammation, and lymphovascular invasion with the histopathological grading of OSCC.

Materials and methods: This study included 30 oral squamous cell carcinoma cases from 2015 to 2021. The surgically operated cases of OSCC were obtained from the archives of the Oral Pathology Department. The subjects were categorized according to the degree of differentiation of OSCC. The parameters like the pattern of invasion of tumour (POI), stromal inflammation, and lymphovascular invasion were assessed and correlated with the different histopathological grades of OSCC.

Results: We observed a statistically significant correlation between the pattern of invasion and stromal inflammation with histopathological grades of OSCC. There was no significant association between lympho-vascular invasion and histopathological grades of OSCC.

Conclusion: We conclude that histopathological parameters like the pattern of invasion and stromal inflammation significantly impact different grades of OSCC. These parameters should be included in routine histo-pathological reports for predicting clinical outcomes and management of the disease. Keywords: invasion, metastasis, tumour front, stromal inflammation, carcinoma

## Introduction

The most prevalent oral cancer, accounting for 80-90% of all malignancies affecting the oral mucosa, is oral squamous cell carcinoma (OSCC) [1]. OSCC is one of the main factors contributing to the rising number of cancer-related fatalities among men in India [2]. Numerous researchers have examined and proposed different prognostic implications for histopathologic and clinicopathologic markers [3,4]. These parameters include the size of the tumour, tumour margins, tumour thickness, the pattern of invasion, eosinophils and stromal contents, chronic inflammatory cells, etc. [5]. Tissue infiltration by cancer at the tumour-front is known as the invasion pattern of the tumour or POI. The American Joint Committee on Cancer (AJCC), 8th Edition, has given five different patterns of tumour invasion (POI). Neoplasia invading in a dispersed or diffused manner is considered more aggressive than invading in a bulky pushing manner [6]. POI significantly determines the prognosis and affects the treatment plan [7].

Inflammation associated with cancer is found to be of great importance in identifying the progression of the neoplasms. The chronic inflammatory infiltration in the stromal response of invading neoplasm has also been favorably associated with patient survival [5]. Three patterns of stromal inflammation are included in the AJCC 8th Edition. A higher proportion of lymphocytes invading the tumour is correlated with an improved prognosis and can be used as a reliable indicator of tumour recurrence [8]. Lymphovascular invasion (LVI), perineural invasion (PNI), and sarcolemmal spread are used to determine aggressive tumour behaviour and predict the disease outcome in OSCC [9]. LVI is a pathologic occurrence where tumour cells invade a vascular or lymphatic vessel's endothelium-lined area without underlying muscle walls [10]. Tumour cell penetration into lymphovascular spaces through the endothelial cell layer is identified as a significant stage in tumour metastasis. It is thought to be a good predictor of some cancers, such as colorectal and prostate cancer [11,12]. Even so, lympho-vascular invasion assessment is still necessary because, according to statistics, its presence in randomly selected tissue sections indicates that many tumour cells are infiltrating the vascular compartment, which raises the possibility of metastasis [13].

The present study aimed to correlate the pattern of tumour-invasion (POI), stromal-inflammation, and lympho-vascular-invasion (LVI) with histopathological-grading of OSCC.

## Materials and methods

**Figure 1:**
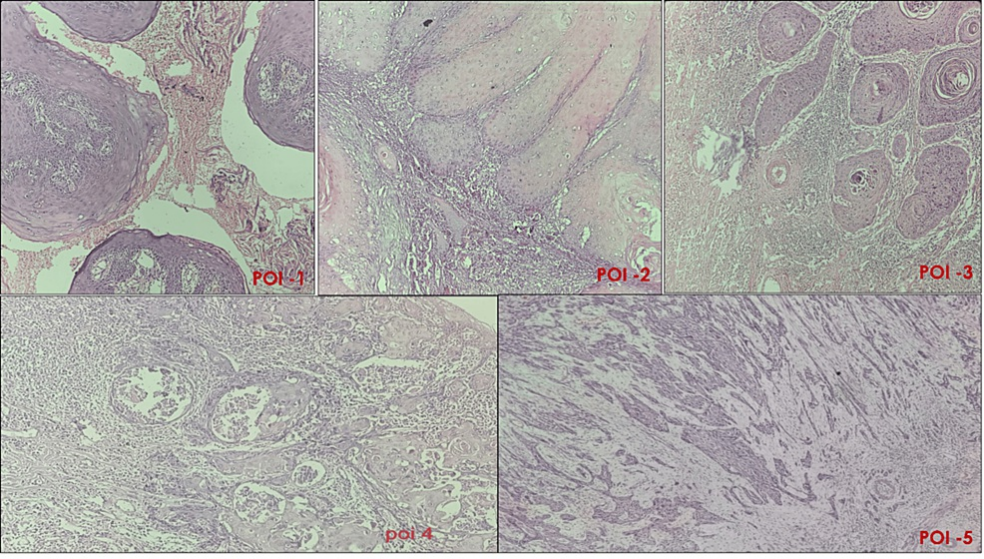
Patterns of Tumor Invasion (POI) (i) Pattern 1- Broad-pushing pattern (ii) Pattern 2 –Pushing-finger pattern/ separate islands of tumour (iii) Pattern 3 – Islands of tumour (more than 15 cells per island) (iv) Pattern 4 – Islands of the tumour with less than 15 cells per island; along with the invasion of single cells (v) Pattern 5 – Satellites of tumour cells with greater than 1 mm distance from the tumour.

This retrospective research was conducted in the Department of Oral and Maxillofacial Pathology, Sharad Pawar Dental College, DMIHER, Sawangi (Meghe), Wardha, Maharashtra, India. Thirty surgically operated cases of OSCC who underwent surgical resection with neck dissection from the year 2015 to 2021 were included in this study. The study began after obtaining prior approval from the Ethical Committee of the Institute (DMIMS (DU)/IEC/2022/291 Dated 05/10/2022). Patients with a history of preoperative radiotherapy or chemotherapy and those with recurrent or distant metastasis were excluded from the research. Clinical parameters such as age, gender, habit history, site, size and clinical appearance of the lesion, and demography were retrieved from the patient records. All of the slides were taken from the department's archive. Three pathologists reviewed the slides in a blinded manner. Based on the histological grading of the carcinomas according to the degree of differentiation, the cases were distributed into three groups: well, moderate, and poorly differentiated SCC. For POI, the invasive tumour front (ITF) was identified. The pattern of invasion was evaluated and classified into different patterns (Table 1, Figure 1).

**Table 1:** Patterns of Tumor Invasion (POI) according to AJCC 8th Edition [4]

Patterns of Tumor Invasion (POI)	
POI 1	Tumor invading in a broad pushing manner.
POI 2	Invasion through large, pushing fingers or discrete, stellate-looking invasive tumor islands.
POI 3	Large islands with more than fifteen cells per island.
POI 4	Tumor islands containing less than fifteen cells each; regardless of the size of the island, this covers invasion by single cells, tumor cell strands, and single-cell filing patterns.
POI 5	A widely distributed tumor infiltration pattern. Any size tumor satellite at the tumor/host interface with at least 1 mm of normal tissue separating it (not fibrosis).

**Figure 2:**
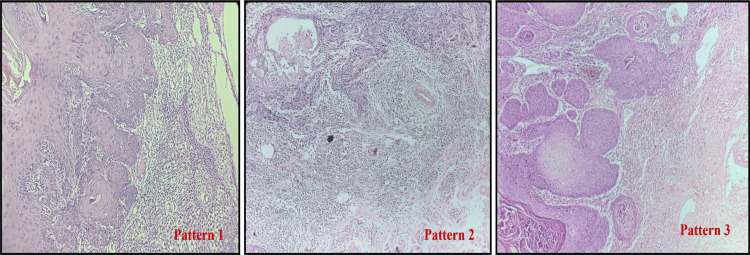
Patterns of Stromal Inflammation (i) Pattern 1 – Dense and continuous rim of lymphoid infiltrate at the invasive tumour front, (ii) Pattern 2 – Lymphoid patches at the invasive tumour front, (iii) Pattern 3 – Minimal or no lymphoid response.

In cases with more than one pattern of invasion, the scoring was done according to the highest pattern present, even if it is present focally (Worst Pattern of Invasion). According to Brandwein-Gensler criteria [4], hematoxylin and eosin-stained slides were evaluated for stromal inflammation/lymphocytic response at the invasive tumour front (ITF) (Table 2, Figure 2).

**Table 2:** Pattern of lymphoid infiltrate/stromal inflammation at the invasive tumour front (ITF) [4]

Pattern of Lymphoid Infiltrate/Stromal Inflammation	
Pattern 1	A dense and continuous rim of lymphoid tissue at the invasive tumor front (ITF).
Pattern 2	Lymphoid infiltrate in patches at the ITF (with discontinuous inflammation along the interface). Any patch of lymphoid infiltrate qualifies as pattern 2 stromal inflammation.
Pattern 3	Minimal or no lymphoid response seen

**Figure 3:**
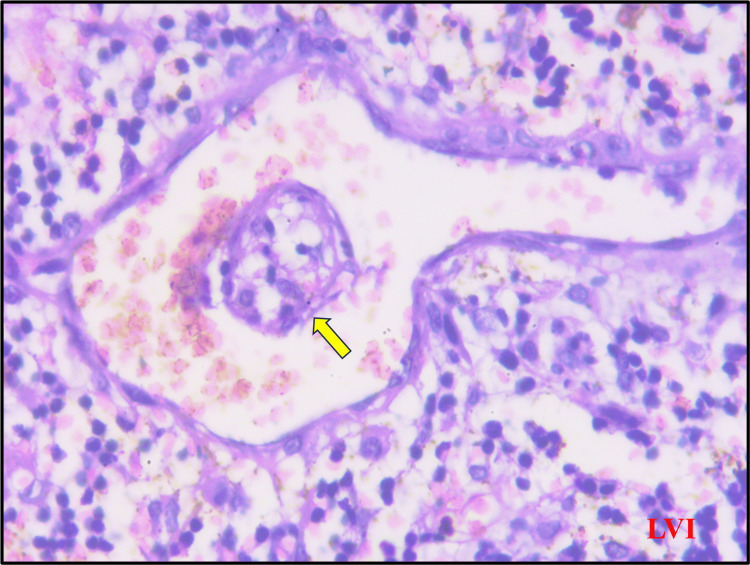
Hematoxylin & eosin section showing lympho-vascular invasion (yellow arrow).

For lympho-vascular invasion, H & E-stained sections were examined for malignant epithelial cells within an endothelial-lined space and marked as positive (Figure 3).

Statistical analysis

The study employed both descriptive and inferential statistics, utilizing the chi-square test. The statistical program used for the research was SPSS 27.0, with a significance level of p<0.05.

## Results

Thirty cases of OSCC were included with a mean age and standard deviation of 52.16 ± 9.71 (29-68 years). On the evaluation of the gender, the majority of the cases were male, 20 (66.66%). In the assessment of histopathological grading, the majority of patients were MDSCC- 23 (76.67%) followed by WDSCC- 6 (20%), and only 1 (3.33%) case was of PDSCC. In our study, a strong association was observed between the histopathological grading of OSCC and the pattern of tumour invasion (POI 3) (P= 0.030) (Table 3).

**Table 3:** Correlation of histopathological grading with Pattern of Invasion PDSCC: Poorly differentiated squamous cell carcinoma; WDSCC: Well-differentiated squamous cell carcinoma; MDSCC: Moderately differentiated squamous cell carcinoma; POI: Pattern of Tumor Invasion; S: Significant

Histopathological Grading	Pattern of Tumor Invasion (POI)					Total	ϗ2-value
	Pattern 1	Pattern 2	Pattern 3	Pattern 4	Pattern 5		
WDSCC	1 (16.67%)	1 (16.67%)	4 (66.67%)	0 (0%)	0 (0%)	6 (20%)	4.70 P=0.030, S
MDSCC	1 (4.35%)	1 (4.35%)	16 (69.57%)	5 (21.74%)	0 (0%)	23 (76.67%)	
PDSCC	0 (0%)	0 (0%)	0 (0%)	1 (100%)	0 (0%)	1 (3.33%)	
Total	2 (6.67%)	2 (6.67%)	20 (66.67%)	6 (20%)	0 (0%)	30 (100%)	

We observed a significant correlation between histopathological grading of OSCC and stromal inflammation (type 2) (P= 0.015) (Table 4).

**Table 4:** Correlation of histopathological grading with stromal inflammation PDSCC: Poorly differentiated squamous cell carcinoma; WDSCC: Well-differentiated squamous cell carcinoma; MDSCC: Moderately differentiated squamous cell carcinoma; S: Significant

Histopathological Grading	Stromal-Inflammation			Total	ϗ2-value
	Pattern 1	Pattern 2	Pattern 3		
WDSCC	5 (83.33%)	1 (16.67%)	0 (0%)	6 (20%)	12.29 p=0.015, S
MDSCC	5 (21.74%)	13 (56.52%)	5 (21.74%)	23 (76.67%)	
PDSCC	0 (0%)	0 (0%)	1 (100%)	1 (3.33%)	
Total	10 (33.33%)	14 (46.67%)	6 (20%)	30 (100%)	

No significant statistical correlation was observed between histopathological grading and lympho-vascular invasion (P= 0.090) (Table 5).

**Table 5:** Correlation of histopathological grading with Lymphovascular Invasion PDSCC: Poorly differentiated squamous cell carcinoma; WDSCC: Well-differentiated squamous cell carcinoma; MDSCC: Moderately differentiated squamous cell carcinoma; LVI: Lymphovascular Invasion; NS: Non-significant

Histopathological Grading	Lymphovascular Invasion (LVI)		Total	ϗ2-value
	Present	Absent		
WDSCC	1 (16.67%)	5 (83.33%)	6 (20%)	0.20 p=0.90, NS
MDSCC	4 (17.39%)	19 (82.61%)	23 (76.67%)	
PDSCC	0 (0%)	1 (100%)	1 (3.33%)	
Total	5 (16.67%)	25 (83.33%)	30 (100%)	

## Discussion

Predominantly occurring in the head-neck region, OSCC is the most frequently occurring malignant tumour of the oral cavity. In 2018, the International Agency for Research on Cancer reported that there were about 354,000 new instances of oral cancer diagnosed worldwide, resulting in 177,000 cancer-related deaths [14]. For OSCC, the overall five-year survival rate is almost 60%, and there hasn't been much of an increase in the last 20 years despite advancements in cancer diagnosis and therapy [15,16]. Additionally, there is an increase in the incidence of OSCC in younger populations [17]. Consequently, risk assessment is valuable for investigating potentially useful markers in OSCC. Significant advancements have been made in cancer research, therapy, evaluation, and management of the disease during the past few decades. It has been repeatedly established that identifying several clinical and histopathological parameters is the most crucial factor in determining the patient prognosis in OSCC. The assessment of histopathological characteristics in tissue sections lays the groundwork for the diagnosis and staging of cancer. In OSCC, metastasis and recurrence are the primary etiological variables that lead to the failure of treatment modality and disease management. Therefore, it is crucial to identify the histological features that either positively or negatively affect the prognosis of oral cancer [4,18].

The prognosis is affected by the tumour's histological differentiation. A worse prognosis is often represented by a severe grade [19]. According to Kolokythas et al., there is a remarkable association between the degree of keratin expression and the grade of differentiation in tongue SCC. Specifically, cases with grade 2 and 3 differentiation showed an increased disease progression by a factor of 6.9 and 11.0, respectively, compared to those with grade 1. Patients with keratin scores of 2 (intermediate) and 3 (least) showed an increase in the progression of the disease by a factor of 2.2 and 6.4 when compared to those having keratin scores of 1 (most) [20]. There are two functional and morphological variations of collective migration found in tumours: the first is characterized by protruding sheets and strands that display evidence of local invasion while remaining in contact with the primary location of the tumour, and the second is characterized by cell clusters (called nests) that are not attached to the tumour-mass and spread through the vasculature and peri-neural structures (the path of least resistance) [21].

In the study conducted by Sethi S et al. [22], the most frequent pattern of invasion was found to be pattern 4 (invasive islands with less than 15 cells per island). They found a significant correlation between the pattern of invasion and survival. No correlation was seen between the pattern of invasion and stromal inflammation. In the current study, invasive islands (>15 cells/island) were the most frequent POI in group I (WDSCC), which was exhibited by 66.67% of the cases; in group II (MDSCC), invasive islands (>15 cells/island) were the predominant POI, shown by 69.57% of cases; and in group III (PDSCC), invasive tumor-islands (less than fifteen cells per island) along with invasion of single-cells were the predominant patterns observed, exhibited by 100% of cases. Our findings are consistent with other studies conducted by Sethi S et al. [22] and Mishra A et al. [23], indicating that the tumour invasion pattern is the most crucial factor in survival. A statistically significant difference (P = 0.030) was observed between the histopathological grading of OSCC and the different patterns of tumor invasion patterns. A substantial prognostic factor based on the pattern of invasion has been documented, which highlights the need to take more extensive biopsies and include the underlying connective tissue stroma. Over the past 20 years, it has been shown that POI, both by itself and in conjunction with weighted scoring systems, can determine a reduced overall survival rate and loco-regional tumour recurrence [7].

It is believed that inflammatory cells infiltrating the tumour and the cytokines they produce (TGF-β, IL-1, and NF-κB) play a significant role in controlling these features [24-27]. In the study done by Sethi S et al. [22], they found no correlation between stromal inflammation and clinical staging of the tumour. However, a significant association was found between stromal inflammation and survival. In this study, 83.33% of group I cases (WDSCC) exhibited a strong inflammatory response (pattern 1 stromal inflammation), 56.52% of group II cases (MDSCC) showed a moderate response (pattern 2 stromal inflammation), and 100% of group III cases (pattern 3 stromal inflammation) exhibited little to no response. The group with moderate differentiation exhibited the greatest mean inflammatory cell count. Our findings are consistent with the research done by Kullage S. et al. [28]. Our investigation found a positive association (P = 0.015) between stromal inflammation and histopathological grading. According to specific theories, angiogenesis, inflammatory cytokines, and inflammatory cells are linked to increased keratinocyte proliferation. These factors may also influence the tumour size, shape, and growth; that is, in situations where there is less of an inflammatory response, the tumours may behave more aggressively clinically [5,27,29].

Due to the fragile basement membrane of the proliferating vasculature channels in an aggressive tumour, malignant neoplastic cells have frequently been observed penetrating the vascular lumens. The difficulties in identifying invasion with certainty have led to several scientists leaving this parameter out when assessing the prognostic implications [24, 30-32]. Additionally, lympho-vascular invasion was seen in 16.67% of patients in group I (WDSCC), 17.39% of patients in group II (MDSCC), and 0% in group III (PDSCC). There was no significant statistical difference between lympho-vascular invasion and histological grading. A study involving five hundred and seventy-one OSCC patients showed that lymphatic invasion was substantially linked to poor disease-specific, disease-free, and overall survival compared to vascular invasion. It was discovered that lymphatic invasion was not a reliable indicator for prognosis [33]. Jardim JF et al. [9] found a significant correlation between lympho-vascular invasion and histopathological grading of OSCC. However, no correlation was seen between lympho-vascular invasion and survival. A combined, comprehensive evaluation of several clinicopathological factors might be a more definitive way to predict the outcome of the disease and determine the treatment required.

The only limitation of this study is the small sample size. A larger sample size would help in obtaining better results. Also, the PDSCC cases are very few compared to those of WDSCC and MDSCC. Including an equal number of cases may provide precise statistical results.

## Conclusions

We conclude that the invasion and stromal inflammation pattern correlate with the histopathological grading of OSCC. Although our study did not show a statistically significant correlation between lymphovascular invasion and histopathological grading of tumours, other studies have shown a different result, and it should be included in routine histopathological reporting. A patient's prognosis is determined by a wide range of factors, which should be taken into consideration while planning appropriate treatment to lower the rates of morbidity and death. These parameters, along with other clinicopathological parameters like tumour depth of invasion, tumour thickness, and tumour budding, should be included in histopathological reporting for better clinical outcomes and to preserve essential data for later research.
